# Tropheryma whipplei [tro-fer’ĭ-mə wi’-pəl-ē-ī]

**DOI:** 10.3201/eid1605.E1605

**Published:** 2010-05

The genus name of this gram-positive, rod-shaped, soil-dwelling bacterium was taken from Greek *trophe* (nourishment) and *eryma* (barrier) because malabsorption was a feature of the infection it caused. The species name honors George Hoyt Whipple (1878–1976), an American pathologist and medical educator, who, in 1907, first described the clinical syndrome later known as Whipple’s disease. In 1991, when sections of the genome were sequenced, the organism was named *T. whippelii*; the spelling was corrected in 2001.
